# Molecular and clinical heterogeneity within *MYC*-family amplified medulloblastoma is associated with survival outcomes: A multicenter cohort study

**DOI:** 10.1093/neuonc/noae178

**Published:** 2024-10-08

**Authors:** Edward C Schwalbe, Janet C Lindsey, Marina Danilenko, Rebecca M Hill, Stephen Crosier, Sarra L Ryan, Daniel Williamson, Jemma Castle, Debbie Hicks, Marcel Kool, Till Milde, Andrey Korshunov, Stefan M Pfister, Simon Bailey, Steven C Clifford

**Affiliations:** Wolfson Childhood Cancer Research Centre, Newcastle University Centre for Cancer, Newcastle upon Tyne, UK; Department of Applied Sciences, Faculty of Health and Life Sciences, Northumbria University, Newcastle upon Tyne, UK; Wolfson Childhood Cancer Research Centre, Newcastle University Centre for Cancer, Newcastle upon Tyne, UK; Wolfson Childhood Cancer Research Centre, Newcastle University Centre for Cancer, Newcastle upon Tyne, UK; Wolfson Childhood Cancer Research Centre, Newcastle University Centre for Cancer, Newcastle upon Tyne, UK; Wolfson Childhood Cancer Research Centre, Newcastle University Centre for Cancer, Newcastle upon Tyne, UK; Wolfson Childhood Cancer Research Centre, Newcastle University Centre for Cancer, Newcastle upon Tyne, UK; Wolfson Childhood Cancer Research Centre, Newcastle University Centre for Cancer, Newcastle upon Tyne, UK; Wolfson Childhood Cancer Research Centre, Newcastle University Centre for Cancer, Newcastle upon Tyne, UK; Wolfson Childhood Cancer Research Centre, Newcastle University Centre for Cancer, Newcastle upon Tyne, UK; Hopp Children´s Cancer Center (KiTZ), Heidelberg, Germany; German Cancer Research Center (DKFZ) and German Cancer Consortium (DKTK), Heidelberg, Germany; Princess Máxima Center for Pediatric Oncology, Utrecht, The Netherlands; University Medical Center Utrecht, Utrecht University, Utrecht, The Netherlands; Hopp Children´s Cancer Center (KiTZ), Heidelberg, Germany; Department of Pediatric Hematology and Oncology, Heidelberg University Hospital, Heidelberg, Germany; National Center for Tumor Diseases (NCT), Heidelberg, Germany; Hopp Children´s Cancer Center (KiTZ), Heidelberg, Germany; Department of Pediatric Hematology and Oncology, Heidelberg University Hospital, Heidelberg, Germany; National Center for Tumor Diseases (NCT), Heidelberg, Germany; Hopp Children´s Cancer Center (KiTZ), Heidelberg, Germany; Department of Pediatric Hematology and Oncology, Heidelberg University Hospital, Heidelberg, Germany; National Center for Tumor Diseases (NCT), Heidelberg, Germany; Wolfson Childhood Cancer Research Centre, Newcastle University Centre for Cancer, Newcastle upon Tyne, UK; Wolfson Childhood Cancer Research Centre, Newcastle University Centre for Cancer, Newcastle upon Tyne, UK

**Keywords:** medulloblastoma, MYC amplification, MYCN amplification, survival

## Abstract

**Background:**

*MYC*/*MYCN* are the most frequent oncogene amplifications in medulloblastoma (MB) and its primary biomarkers of high-risk (HR) disease. However, while many patients’ *MYC(N)*-amplified tumors are treatment-refractory, some achieve long-term survival. We therefore investigated clinicobiological heterogeneity within *MYC(N)*-amplified MB and determined its relevance for improved disease management.

**Methods:**

We characterized the clinical and molecular correlates of *MYC*- (*MYC*-MB; *n* = 64) and *MYCN*-amplified MBs (*MYCN*-MB; *n* = 95), drawn from >1600 diagnostic cases.

**Results:**

Most *MYC*-MBs were molecular group 3 (46/58; 79% assessable) and aged ≥3 years at diagnosis (44/64 [69%]). We identified a “canonical” very high-risk (VHR) *MYC*-amplified group (*n* = 51/62; 82%) with dismal survival irrespective of treatment (11% 5-year progression-free survival [PFS]), defined by co-occurrence with ≥1 additional established risk factor(s) (subtotal surgical-resection [STR], metastatic disease, LCA pathology), and commonly group 3/4 subgroup 2 with a high proportion of amplified cells. The majority of remaining noncanonical *MYC*-MBs survived (i.e. non-group 3/group 3 without other risk features; 11/62 (18%); 61% 5-year PFS). *MYCN* survival was primarily related to molecular group; *MYCN*-amplified SHH MB, and group 3/4 MB with additional risk factors, respectively defined VHR and HR groups (VHR, 39% [35/89]; 20% 5-year PFS/HR, 33% [29/89]; 46% 5-year PFS). Twenty-two out of 35 assessable *MYCN*-amplified SHH tumors harbored *TP53* mutations; 9/12 (75%) with data were germline. *MYCN*-amplified group 3/4 MB with no other risk factors (28%; 25/89) had 70% 5-year PFS.

**Conclusions:**

*MYC(N)*-amplified MB displays significant clinicobiological heterogeneity. Diagnostics incorporating molecular groups, subgroups, and clinical factors enable their risk assessment. VHR “canonical” *MYC* tumors are essentially incurable and SHH-*MYCN*-amplified MBs fare extremely poorly (20% survival at 5 years); both require urgent development of alternative treatment strategies. Conventional risk-adapted therapies are appropriate for more responsive groups, such as noncanonical *MYC* and non-SHH-*MYCN* MB.

**Key Points**

- *MYC*(*N*)-amplified medulloblastoma is clinically and biologically heterogeneous.
- “Canonical” *MYC* and SHH-*MYCN* are near incurable and require new approaches.
- Remaining *MYC*(*N*) patients commonly survive and may be stratified for conventional therapies.

**Importance of the Study**

Medulloblastoma (MB) is among the most common malignant brain tumors of childhood. *MYC(N)* family amplifications (*MYC*, ~3%; *MYCN*, ~6% of tumors) are the primary molecular biomarkers of poor prognosis, high-risk (HR) disease, underpinning risk-stratified therapies in international, biomarker-driven clinical trials (e.g. SIOP-PNET5-MB, SIOP-HR-MB, and SJMB12). Previous clinical trial analyses have indicated outcome differences within *MYC/N* amplified MB; however, studies to understand this heterogeneity have previously been limited by their rarity. We assembled a cohort of 64 *MYC* and 95 *MYCN*-amplified tumors and established significant clinicobiological heterogeneity within both *MYC* and *MYCN*-amplified disease. Disease molecular group is the primary determinant of their clinical features, interacting with other risk factors to define prognosis. We identify, and proffer clinico-molecular risk stratification schema for, very HR tumor groups (“canonical” *MYC* and SHH/*MYCN*) in which current multimodal therapies are ineffective, and HR groups compatible with long-term survival. This heterogeneity must be considered diagnostically and has the potential to immediately impact clinical management.

Medulloblastoma (MB) is one of the most common malignant brain tumors of childhood. Approximately 30% of patients will die of their disease, while survivors commonly experience life-long disease and treatment-associated morbidities.^1^ Focal amplifications of *MYC* or *MYCN* are the most frequent oncogenic amplifications, and have been consistently associated with a poor prognosis across different clinical studies.^2–8^ This has led to their routine diagnostic assessment as the primary biomarkers of high-risk (HR) MB disease, underpinning risk-stratified therapies in international biomarker-driven clinical trials (e.g. SIOP-PNET5-MB; NCT02066220,^9^ SIOP-HR-MB; NCT pending,^10^ SJMB12; NCT01878617).

However, retrospective survival analyses of the SIOP-UKCCSG-PNET3 and HIT-SIOP-PNET4 trial cohorts demonstrated outcome differences within *MYC(N)*–amplified MB, suggesting *MYC(N)* amplification in the absence of other clinicopathological risk factors may not confer a poor prognosis,^4,11^ leading to such patients potentially incurring unnecessary side effects from intensified risk-adapted protocols. Conversely, *MYC(N)* amplification in conjunction with large-cell/anaplastic (LCA) histology has long been recognized to confer poor prognosis.^6,12^

*MYC(N)*-amplified MBs are molecularly heterogeneous, which may influence their clinical behavior. MB comprises 4 consensus molecular groups: WNT (MB_WNT_), SHH (MB_SHH_), and non-WNT/non-SHH (comprising groups 3 and 4 [MB_Grp3_, MB_Grp4_]).^13–15^*MYC* amplifications occur predominantly in MB_Grp3_ but are observed to a lesser extent in all other groups.^7,16^ In contrast, *MYCN* amplifications are mainly found in MB_SHH_ and MB_Grp4_.^16–19^*MYCN*-amplified SHH MB is associated with *TP53* mutation, commonly in the germline,^20^ chromothripsis, and a poor prognosis.^17,21^ Conversely, *MYCN* amplification does not associate with prognosis in MB_Grp4_.^2,17,22^ Indeed, *MYCN-*amplified group 4 MB with no other HR disease features were treated as standard risk in the SIOP-PNET5 clinical trial.^9^

Recent studies have identified further heterogeneity of potential prognostic significance to *MYC(N)-*amplified tumors; these include the identification of component molecular subgroups within each molecular group^17,23–25^ which are detected using DNA methylation microarray, alongside variations in the pattern and proportion of cells displaying *MYC(N)* amplification.^4^

Understanding differences in molecular pathology and clinical behavior within the *MYC(N)*-amplified group of MBs is thus essential to define their optimal clinical management. However, their relative rarity (~3% [*MYC*] and ~6% [*MYCN*] of all MBs) has limited investigations to small numbers (i.e. typical *n* < 10 per study) in clinical trials and research studies published to date. To address this, we assembled a retrospective cohort of 64 *MYC* and 95 *MYCN*-amplified tumors, derived from screening approximately 1600 MBs, representing the largest cohorts studied to date. We report a comprehensive characterization of their clinical features, molecular pathology, and survival outcomes, revealing significant clinically relevant heterogeneity, including very high-risk (VHR) tumor groups near-universally refractory to current therapies, and groups associated with significant long-term survival. These findings serve as a foundation to (i) immediately aid the clinical interpretation of contemporary molecular diagnostics, and (ii) inform the design of future clinical and research investigations, for this important tumor group.

## Materials and Methods

### Study Design and Participants

Tumor samples were provided by the UK CCLG (CCLG-approved biological study BS-2007–04); informed, written consent was obtained. Samples were also obtained from retrospective, previously published, international Heidelberg cohorts.^26,27^ The *MYC*-amplified cohort comprised 34 patients from the CCLG and 30 from Heidelberg; 57 patients in the *MYCN*-amplified cohort were drawn from the CCLG and 38 from Heidelberg. Tumor investigations were done with approval from Newcastle-North Tyneside Research Ethics Committee (reference 07/Q0905/71); all tumor material was collected in accordance with this approval.

**Figure 1.**
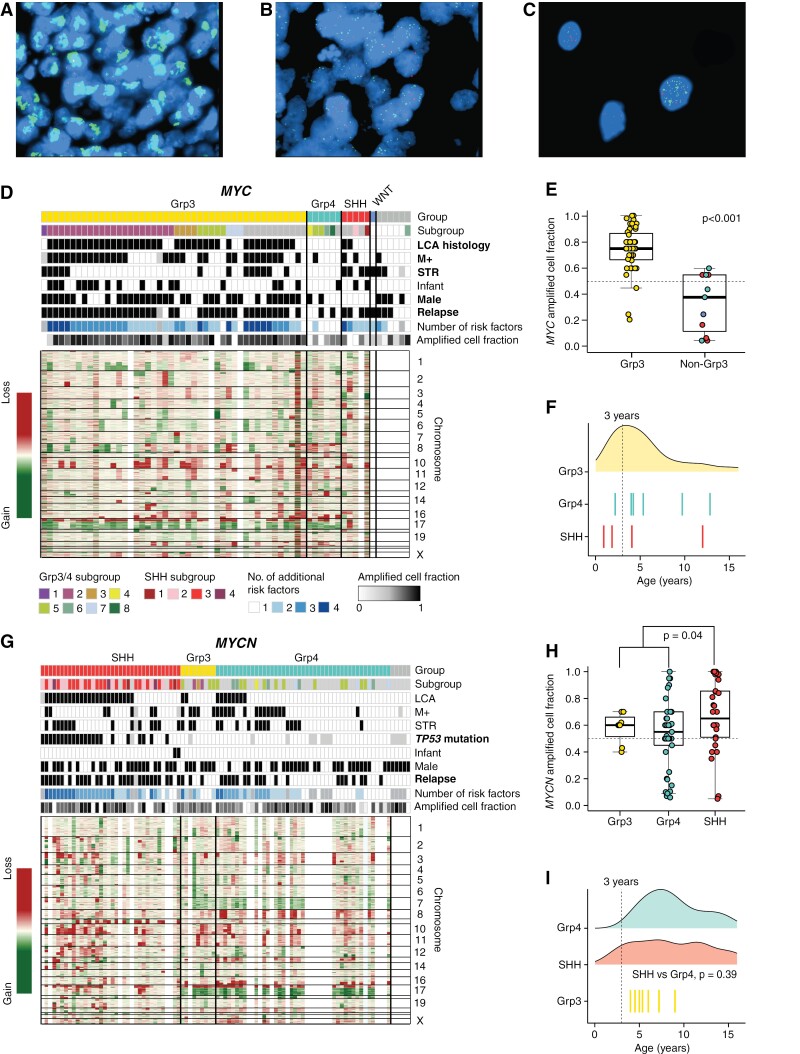
Clinicopathological and molecular features of *MYC*-MB and *MYCN*-MB. (A) Interphase fluorescence in situ hybridization (iFISH) of a group 3 tumor showing high levels of *MYC* amplification (green) vs centromeric control (red) in the majority of cells. (B, C) Example of a *MYC*-amplified group 4 tumor with a mixture of *MYC*-amplified, *MYC* gained and normal cells at 40× and 100× magnification. Clinical, molecular, and cytogenetic features are shown for *MYC*-MB (D) and *MYCN*-MB (G), arranged by molecular group. Groups (SHH, red; group 3, yellow; group 4, green; unknown, gray) and subgroups are colored by convention. Missing data are shown in gray. Factors with significant enrichment in specific molecular groups are shown in bold text (<.05, Fisher’s Exact test). The relationship between amplified cell fraction and molecular group is shown for *MYC*-MB (E) and *MYCN*-MB (H). Age distribution is shown for *MYC*-MB (F) and *MYCN*-MB (I). For molecular groups with few members, individual data points are shown.

No statistical methods were used to predetermine the sample size. We interrogated our retrospective tumor cohorts to identify patients with *MYC*-MB (*n* = 64) and *MYCN*-MB (*n* = 95). Amplification was identified by iFISH (fluorescence in situ hybridization) and/or copy number (CN) estimates from microarray (methylation or SNP6). The criteria for identifying *MYC*-MB and *MYCN*-MB by FISH have been described previously.^4^ Briefly, for each assessable tumor, 100–200 nonoverlapping nuclei were examined, enabling the proportion of amplified cells to be estimated. Individual cells were defined as amplified if the ratio of test probe:centromeric control ratio exceeded >4:1). Individual tumors were classed as amplified when they contained (1) amplification in ≥5% of cells and (2) evidence of cells with a “speckled” or “clumped” signal patterns consistent with double minute formation or homogeneously staining regions (Figure 1). Amplifications from the SNP6 array were called as previously described^16^; for calling amplifications from 450k/EPIC methylation arrays, CN was derived using conumee v4.2 and amplifications at the *MYC*/*MYCN* loci defined as being focal (<10 Mb), with amplitude >0.4.

Amplification was identified using published criteria^4^ by iFISH (fluorescence in situ hybridization) and/or CN estimates (Illumina 450k/EPIC methylation or Affymetrix SNP6 microarrays^16^). For calling amplifications from 450k/EPIC methylation arrays, CN was derived using conumee v4.2, and amplifications at the *MYC*/*MYCN* loci were defined as being focal (<10 Mb), with amplitude >0.4.

### Biological Characterization *of MYC*-MB and *MYCN*-MB

The principal molecular group was assigned using Illumina methylation arrays or by MS-MIMIC for low-quantity and/or poor-quality samples as previously described.^17,28–30^ For samples with 450k/EPIC arrays, the molecular group and group 3/4 subgroup were assigned using MNPv11b4 https://www.molecularneuropathology.org/mnp/.^28^ SHH subgroup was assessed as described.^31^

We assessed established MB clinical, pathological, and molecular features for their associations with *MYC(N)*-amplified disease. Histopathological variants were assigned according to WHO 2021 guidelines^14^; all tumors were centrally reviewed. Metastatic status was assigned using Chang’s criteria^32^; M0/1 disease (M−) was compared against M2/3 disease (M+). Tumors were designated subtotally resected (STR) if their postsurgical residuum exceeded 1.5 cm^2^.^33^

*TP53* and additional MB mutations were identified as previously described.^22,34^ Chromosomal abnormalities and amplifications of *GLI1* and *GLI2* were identified by analysis of CN profiles and/or iFISH. Chromothripsis was inferred from SNP6 microarray-based DNA CN profiles, according to previously described criteria.^20,23,35,36^

Gene fusions were detected from RNA-seq data as previously described.^37^ Primer sequences for confirmation of fusion events by RT-PCR are shown in Supplementary Figure 4.

### Statistical Analysis

Survival analysis was performed using progression-free survival (PFS), defined as the interval between diagnosis (i.e. date of surgery) and disease progression (defined as the time at which disease progression was confirmed by MRI). While PFS/OS was available for almost every *MYC*-amplified tumor (63/64 and 64/64 tumors, respectively), OS was less widely available (94/95 and 84/95 PFS/OS) for *MYCN-*amplified tumors. There was no significant difference between OS and PFS in either cohort (Supplementary Figure 1); consequently, we used PFS for subsequent survival analyses. Kaplan-Meier curves were plotted and differences in survival between groups were assessed using log-rank tests. Univariable Cox models were constructed for key disease features and proportionality of hazards confirmed by examining scaled Schoenfeld residuals. We assessed the prognostic utility of current molecular and clinical variables. Fisher’s exact and chi-squared tests were used to assess associations between categorical variables. ANOVA and *t*-tests were used to compare continuous variables between groups. Significant associations were defined as having *P* < .05. Statistical and bioinformatics analyses were done using R statistical environment (version 4.3.0),^38^ using the survival v3.5-5, and rms v6.7-0 packages.

## Results

### Detection of *MYC*(*N*)-Amplified Tumors

*MYC*-MB and *MYCN*-MB (*n* = 64 and 95, respectively; Table 1) were identified by iFISH and/or microarray analysis, with the majority (116/159; 73%) assessed by both methods. Despite strong concordance overall, some tumors with lower percentages of amplified cells by iFISH (*MYC*-MB tumors, *n* = 5/49 [10%] tumors with both iFISH and methylation-array derived call, 7%–25% cells amplified; *MYCN*-MB tumors, *n* = 7/62 (11%) tumors with both iFISH and methylation-array derived call, 7%–60% cells amplified), were not detectable by CN array. Thus, while assessment of *MYC(N)*-amplification is readily accessible from DNA methylation microarrays, superior sensitivity together with the reported clinical significance of lower amplification frequencies,^4^ mandates continued use of iFISH as the “gold standard” for clinical assessment.^39^

**Table 1.** Demographics and clinical characteristics of *MYC*-MB and *MYCN*-MB cohorts.

	*MYC* (*n* = 64)	*MYCN* (*n* = 95)	*P* value
Sex			
Male	39(61%)	62(65%)	
Female	25(39%)	33(35%)	.62
Age at diagnosis			
Median, years (range)	4.6 (0.9–15.8)	8.0 (1.9–33.3)	
<2.99	20(31%)	2 (2%)	
>3.00	44(69%)	93(98%)	<.001
Histopathological variant			
LCA	40(63%)	33(35%)	
Classic	23(36%)	57(61%)	
DN	1 (1%)	4 (4%)	
MB-NOS	0	1	**.002**
Metastatic stage			
M−	28(45%)	27(71%)	
M+	34(55%)	65(29%)	.06
No data	2		
Resection			
STR	20(32%)	30(32%)	
GTR	43(68%)	64(68%)	1
No data	1	1	
Isochromosome17q			
Present	26(44%)	41(46%)	
Absent	32(56%)	48(54%)	1
No data	6	6	
*TP53* mutation			
Present	1 (2%)	21(25%)	
Absent	51(98%)	62(75%)	**<.001**
No data	12	12	
*GLI1/2* amplification			
Present	0 (0%)	11(15%)	
Absent	55(100%)	64(85%)	**.002**
No data	9	20	
Molecular group			
WNT	1 (2%)	0 (0%)	
SHH	5 (9%)	36(40%)	
Group 3	46(79%)	10(11%)	
Group 4	6 (10%)	45(49%)	**<.001**
No data	6	4	
Subgroup—group 3/4			
1	1 (2%)	0 (0%)	
2	22(54%)	2 (7%)	
3	4 (10%)	1 (4%)	
4	1 (2%)	1 (4%)	
5	7 (17%)	14(52%)	
6	2 (5%)	5 (19%)	
7	3 (7%)	4 (15%)	
8	1 (2%)	0 (0%)	
No data	12	27	
Subgroup—SHH			
1	1 (50%)	2 (6%)	
2	1 (50%)	3 (9%)	
3	0	18(55%)	
4	0	10(30%)	
No subgroup data	3	3	
Treatment			
RTX alone at diagnosis	5 (8%)	5 (6%)	
CTX alone at diagnosis	21(36%)	4 (4%)	
RTX and CTX at diagnosis	32(54%)	83(90%)	
None	1 (2%)	0 (0%)	**<.001**
No data	5	3	
Follow-up time			
Median, years (range)	6.2 (0.1–17)	6.3 (0.1–14)	

PFS was available for 63/64 *MYC*-amplified patients and for 94/95 *MYCN*-amplified patients. *P* values from Fisher’s exact tests are shown. *P* values <.05 are shown in bold text.

### Molecular Groups and Subgroups

*MYC*-MB tumors were predominantly MB_Grp3_ (46/58, 79%; Table 1; Figure 1D), although appreciable numbers were also observed in MB_SHH_ (*n* = 5, 9%) and MB_Grp4_ (*n* = 6, 10%). Within *MYC*-MB_Grp3_, subgroup 2 was most prevalent (22/41, 54% assessable tumors). *MYCN*-MB tumors were typically MB_SHH_ and MB_Grp4_ (36/90 [40%] and 45/90 [49%], respectively). *MYCN-*MB_SHH_ were primarily members of MB_SHH_ subgroups 3 and 4; *MYCN-*MB_Grp4_ were subgroups 4–7 where data was available (Table 1; Figure 1G).

### Clinicopathological Characteristics and Subclonal Amplification

Specific clinicopathological disease features were strongly associated with molecular group identity in both *MYC* and *MYCN*-MB (Table 1). Infants (<3.0 years) and younger children (3.0–4.99 years) predominated in *MYC*-MB (31% <3 years; median age at diagnosis 4.6 years). In contrast, only 2/95 (2%) patients with *MYCN*-MB were <3 years (Table 1; Figure 1G, I). The predominance of SHH subgroups 3 and 4 within *MYCN*-MB_SHH_ was consistent with their noninfant age profile.^40^ Male sex was significantly enriched in *MYC*-MB_Grp3_ (33/46 [72%] *MYC*-MB_Grp3_ vs 2/12 [17%] *MYC*-MB_non-Grp3_, *P* = .0008; Figure 1D) and also predominated in *MYCN*-MB (Figure 1G), regardless of molecular group (1.88:1 M:F ratio vs 1.5:1 typically observed disease wide^1^).

Most (52/63; 83%) *MYC*-MB presented with ≥1 additional clinicopathological risk factor (Figures 1D and 3F). The majority of *MYC-*MB_Grp3_ tumors had LCA pathology (38/46 [83%] *MYC-*MB_Grp3_ vs 2/12 [17%] *MYC-*MB_non-Grp3_; *P* < .0001)_._ Notably, there were no LCA *MYC-*MB_Grp4_ tumors (0/6; Figure 1D). In addition, *MYC-*MB_Grp3_ tumors were significantly enriched for metastatic disease compared to *MYC-*MB_non-Grp3_ (30/44 [68%] vs 3/12 (25%); *P* = .018). The majority of *MYC-*MB_Grp3_ tumors exhibited high proportions of *MYC*-amplified cells by iFISH, in contrast to *MYC*-MB_non-Grp3_ (mean cells amplified 74% vs 33%; *P* < .0001; Figure 1E). Albeit with small numbers of assessable tumors, subtotal resection (STR) was a feature of most (4/5) *MYC-*MB_SHH_ tumors (Figure 1D).

Fewer (56/91; 62%) *MYCN*-MBs presented with ≥1 other clinicopathological risk factor (Figures 1G and 4F). *MYCN-*MB_SHH_ were also strongly associated with LCA pathology (23/35 [66%] vs 10/54 [19%] in *MYCN-*MB_Grp3/4_, *P* < .0001, Figure 1G). *MYCN-*MB_SHH_ similarly had a significantly increased proportion of amplified cells (mean 67% vs 54% in *MYCN-*MB_Grp3/4_; *P* = .04; Figure 1H). STR and M+ disease appeared equivalently distributed across *MYCN-*MB_SHH_ and *MYCN-*MB_Grp3/4_ (Table 1)_._

### Genomic Profiles

*MYC-*MB mutational (*n* = 22; Supplementary Figure 2) and CN profiles (*n* = 53; Figure 1D) were consistent with MB_Grp3_ and MB_Grp4_ more widely.^17,25^ Additional gene-specific driver mutations were uncommon (Supplementary Figure 2). In contrast, *MYCN*-MB_SHH_ (*n* = 30 CN/*n* = 18 mutation profiles) and *MYCN*-MB_Grp3/4_ (*n* = 37 CN/*n* = 13 mutation) harbored distinct CN and mutation profiles (Figure 1G; Supplementary Figure 3). Within *MYCN-*MB_SHH_, 9q loss was common (14/30, 47%). In contrast, i17q was common (29/37, 78%) in *MYCN-*MB_Grp3/4_*. TP53* mutations were detected in 22/35 (63%) of assessed *MYCN-*MB_SHH_ tumors (missense, *n* = 16/19 with available information; frameshift, *n* = 3/19), but not in *MYCN-*MB_Grp3/4_ (*P* < .0001). The majority (18/22, 82%) of *TP53* mutations within *MYCN*-MB_SHH_ co-occurred with 17p loss (*P* = .00059) and most were germline (9/12 [75%] with available data). *TP53* mutations occurred in all *MYCN*-MB_SHH_ subgroups, but most prevalently in subgroups 3 and 4 (respectively, 10/18, 56% and 9/10, 90% assessable tumors). *GLI2* (10/35, 29%) or *GLI1* (1/35, 3%) amplifications were associated with *MYCN-*MB_SHH_, and only found in *TP53* mutated tumors (*P* = .0045; Supplementary Figure 3).

### Genomic Instability Patterns: Chromothripsis and RNA Fusion Transcripts

**Figure 2.**
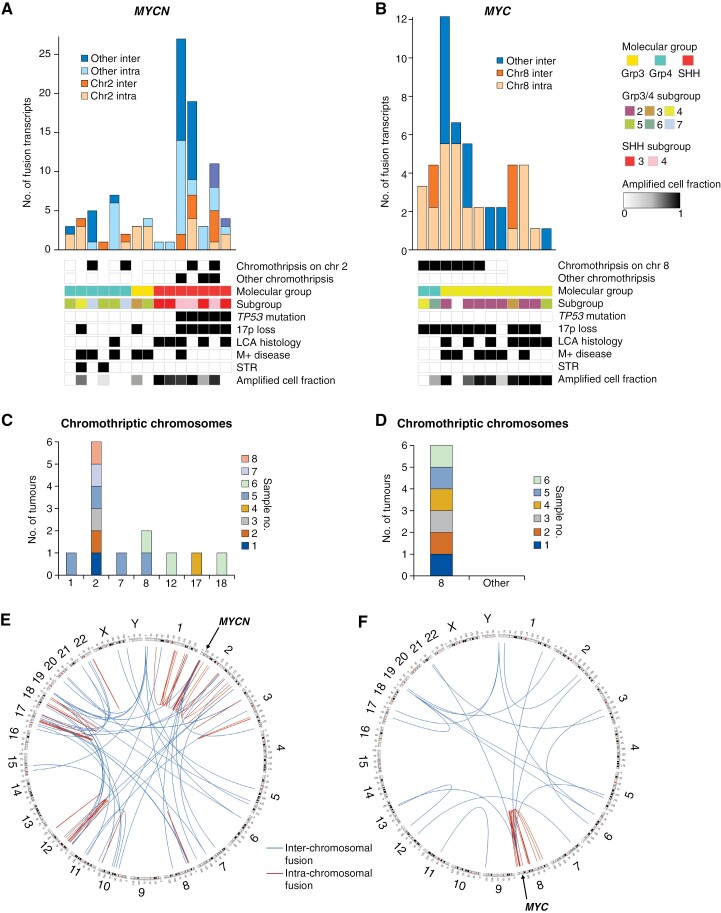
Differential patterns of chromothripsis and fusion transcripts within *MYC*-MB and *MYCN*-MB cohorts. Type and frequency of RNA fusion transcripts in (A) *MYCN* and (B) *MYC*-amplified tumors with molecular group, subgroup, chromothriptic chromosomes, and *TP53* mutated status indicated. Missing data are shown by an empty cell. Chromosomal distribution of chromothripsis is shown for 8 individual *MYCN*-amplified tumors (C) and 6 individual *MYC*-amplified tumors (D), with CN profiles from SNP6 array data (each tumor individually colored within each cohort). Circos plots show the distribution of RNA fusion transcripts in (E) *MYCN* (*n* = 15, data combined) and (F) *MYC*-amplified tumors (*n* = 12, data combined); interchromosomal fusions shown in blue and intrachromosomal fusions shown in red.

We next assessed patterns of CN, chromothripsis, and gene fusion events in our cohorts. Chromothripsis was common in both *MYCN*-MB (8/23 [35%] assessable tumors) and *MYC*-MB (6/11 [55%]), but its patterns and correlates were markedly different. In *MYCN*-MB, chromothripsis was found in both *MYCN-*MB_SHH_ and *MYCN-*MB_Grp4_ (6/14 vs 2/9; *P* = .40), co-occurred with *TP53* mutation in *MYCN-*MB_SHH_ (6/8), and manifested in multiple chromosomes (Figure 2A; Supplementary Figure 4A). In contrast, chromothripsis in *MYC*-MB occurred in conjunction with 17p loss (6/6), without *TP53* mutation (5/5 assessable), and was restricted to chromosome 8 (*MYC* at 8q24; Figure 2B).

The RNA fusion transcript landscape (*n* = 27 tumors) further supported differential genomic instability patterns. Thirty-two putative oncogenic gene fusions were identified (*n* = 23, *MYC*-MB; *n* = 9, *MYCN*-MB). Seven out of 10 (*MYC*-MB) and 6/8 (*MYCN*-MB) gene fusion loci assessed validated successfully (RT-PCR/Sanger sequencing; Supplementary Figure 4). Consistent with chromothripsis patterns, *MYCN*-MB had fusions affecting many chromosomes (Figure 2A, C; Supplementary Figure 4), while *MYC-MB* exhibited intrachromosomal fusions only on chromosome 8 (Figure 2B, D). Fusions involved chromosomes showing evidence of chromothripsis or multiple segmental changes, (Figure 2E, F; Supplementary Figure 4), and genes within coamplified regions.

*MYCN*-MB fusion transcripts were unique to each tumor; 2 recurrent fusion-partner genes, *DDX1* and *NBAS* (immediately upstream of *MYCN*^41^) were involved in fusions with each other and additional partners (*MYCNOS*) in 3 *MYCN-*MB_Grp3/4_ tumors, but fusion position and gene order were not conserved (Supplementary Figure 4B). In *MYC*-MB, fusion transcripts involving *PVT1* were most common (7/12 *MYC*-MB tumors; Figure 2F; Supplementary Figure 4C), were exclusive to *MYC*-MB (vs 220 non-*MYC*-MB tumors^37^) and present in both *MYC-*MB_Grp3_ and *MYC-*MB_Grp4_.^16^

### Outcome Differences in *MYC*(*N*)-Amplified MB: Clinical and Molecular Correlates

**Figure 3.**
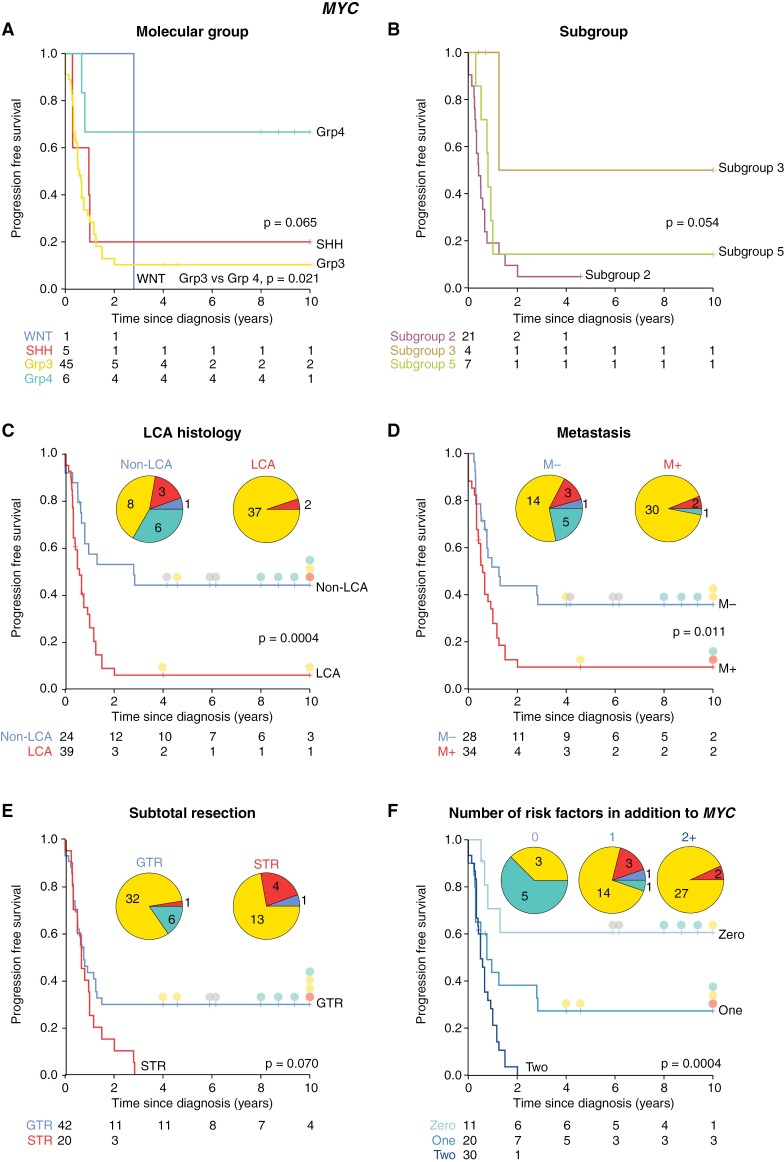
Survival of patients with *MYC*-amplified tumors by clinical and molecular risk features. (A–F) Kaplan-Meier plots and at-risk tables are shown for *MYC*-amplified tumors. Where appropriate, the molecular group is indicated by filled circles adjacent to censor points for survivors with PFS ≥ 4 years; the molecular group is shown on inset pie charts. Certain *MYC*-amplified tumors lacked molecular group information and were omitted from the pie charts. Molecular group colors: SHH, red; group 3, yellow; group 4, green; N/A, gray.

In *MYC*-MB, *MYC-*MB_Grp3_ had significantly worse survival than *MYC-*MB_Grp4_ (*P* = .010; Figure 3A; Supplementary Figure 5); *MYC*-associated disease progression and/or all relapses typically occurred rapidly within 2 years of initial diagnosis. However, long-term survivors were observed in all non-MB_WNT_ groups. Survival was dismal within *MYC-*MB_Grp3_ subgroup 2, with 20/21 patients showing relapse or disease progression within 2 years of diagnosis (5-year PFS 5%; *P *= .054, Figure 3B; Supplementary Figure 5). Moreover, survival for *MYC-*MB_Grp3_ was not dependent on infant status (*P* = .08; Supplementary Figure 5E). The behavior of other *MYC*-MB_Grp3/4_ subgroups remains unclear, due to small sample numbers, however, subgroup 5 patients (*n* = 7) also showed rapid relapse and poor PFS, with 6/7 relapsing or progressing within 2 years of diagnosis. Likewise, LCA pathology conferred a significantly poorer prognosis (5-year PFS 6%; *P* = .0004, Figure 3C; Supplementary Figure 5). The LCA *MYC*-MB group comprised both infants (*n* = 13), most of whom (11/13 (86%)) received no upfront radiotherapy, and older children (*n* = 26, 22/25 of whom received high-dose radiotherapy); this latter group contained the only 2 long-term survivors (Figure 3C). M+ disease was also significantly associated with worse survival (*P* = .011, Figure 3D; Supplementary Figure 5), whereas subtotal resection was not prognostic (Figure 3E). The strongest univariable survival predictor within *MYC-*MB was the percentage of amplified cells (HR 11.9, 95%CI 3.01–47.3, *P* = .0004; Supplementary Figure 5), which was significantly higher in *MYC-*MB_Grp3_ (Figure 1E). Overall, *MYC-*MB_Grp3_ long-term survivors (i.e. ≥4 years postdiagnosis) were characterized by an absence of additional risk factors (i.e. STR/M+/LCA; Figure 3F).

**Figure 4.**
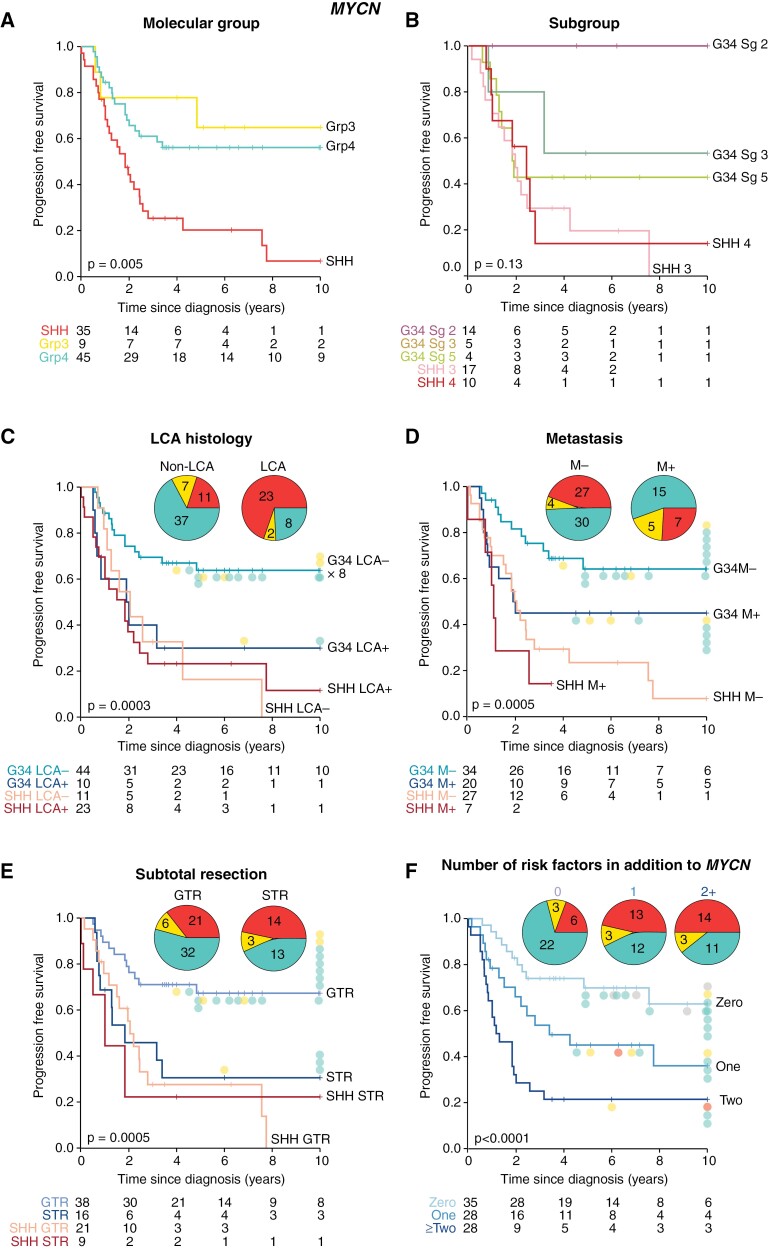
Survival of patients with *MYCN*-amplified tumors by clinical and molecular risk features. (A–F) Kaplan-Meier plots and at-risk tables are shown for *MYCN*-amplified tumors. Where appropriate, the molecular group is indicated by filled circles adjacent to censor points for survivors with PFS ≥ 4 years; the molecular group is shown on inset pie charts. Certain *MYCN*-amplified tumors lacked molecular group information and were omitted from the pie charts. Molecular group colors: SHH, red; group 3, yellow; group 4, green; N/A, gray.

Within *MYCN*-MB, *MYCN*-MB_SHH_ was associated with very poor survival (5-year PFS 20%; *P* = .005, Figure 4A; Supplementary Figure 5); survival in all assessable SHH subgroups (3 and 4) was equivalently poor (Figure 4B). The median time to relapse for *MYCN*-MB_SHH_ was 1.4 years (range 0.1–7.8). *MYCN*-MB_Grp4_ had significantly better outcomes than *MYCN*-MB_SHH_ (5-year PFS 56% vs 20% respectively; *P* = .0005) and, while *MYCN*-MB_Grp3_ were less common (*n* = 9/90), this group had survival rates comparable with *MYCN*-MB_Grp4_ (5-year PFS 65%; *P* = .58; Figure 4A). Molecular features significantly associated with poorer prognosis in univariable analyses included the SHH group and strongly SHH-associated features (e.g. *TP53* mutation, *GLI1/2* amplification; Supplementary Figure 5); neither feature was associated with a significantly different PFS within the *MYCN*-MB_SHH_ cohort (Supplementary Figure 5). The prognostic significance of HR disease features within *MYCN*-MB was molecular group dependent. The presence/absence of established risk features (M+, LCA, and STR) was prognostic within *MYCN*-MB_Grp3/4_ (Figure 4C–E); in contrast, the *MYCN*-MB_SHH_ group had a poor outcome regardless of other HR features, defining a VHR group in its own right. Overall, long-term survivors (i.e. ≥4 years postdiagnosis) were characterized by MB_Grp3/4_ disease with an absence of additional risk factors (Figure 4F).

An additive interaction between *MYC(N)*-amplification and additional clinicopathological risk factors has been suggested previously.^4^ Patients with *MYC*-MB, but otherwise standard risk, achieved 5-year PFS of 61%; 5-year PFS reduced to 29% with one additional risk factor (M+/LCA/STR) with no long-term survivors harboring ≥2 additional risk factors (Figure 3F). Patients with *MYCN-*MB and no additional risk factors had 5-year PFS of 70%; where molecular group was known, all long-term survivors (≥4 years postdiagnosis; *n* = 13) were MB_Grp3/4_. In contrast, patients with one additional risk factor had 45% 5-year PFS (7/8 long-term survivors were MB_Grp3/4_), and patients with ≥2 additional risk factors had 21% 5-year PFS (Figure 4F).

### Cranio-Spinal Irradiation Is Ineffective in *MYC*-MB With Additional Risk Factors

**Figure 5.**
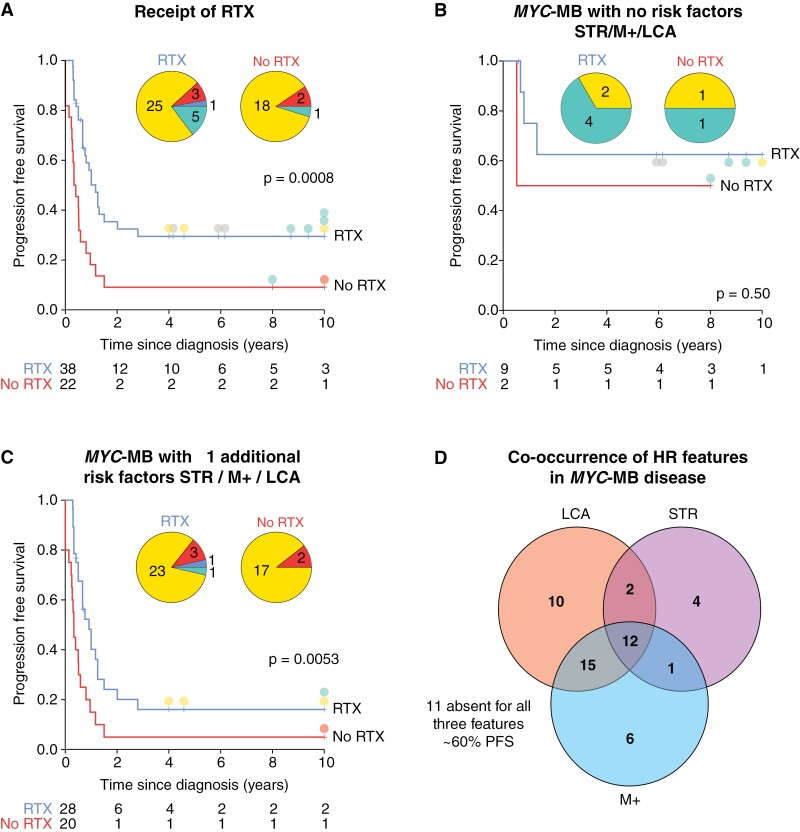
Cranio-spinal irradiation is ineffective in *MYC*-amplified tumors with additional established risk features. (A) Survival of *MYC*-amplified tumors by receipt of radiotherapy. (B) Survival of nonmetastatic, gross-totally resected, non-LCA *MYC*-amplified tumors, stratified by receipt of radiation. (C) Survival of *MYC*-amplified tumors with positivity for one or more HR features in addition to *MYC* amplification, stratified by receipt of radiation. Where appropriate, the molecular group is indicated by filled circles adjacent to censor points for survivors with PFS ≥ 4 years; the molecular group is shown as inset pie charts; certain *MYC*-amplified tumors lacked molecular group information and were omitted from pie charts. (D) Venn diagram summarizes co-occurrence of HR disease features in *MYC*-MB. Molecular group colors: SHH, red; group 3, yellow; group 4, green; N/A, gray.

Overall, receipt of upfront cranio-spinal irradiation (CSI) was associated with significantly improved survival in *MYC*-MB patients (5-year PFS 30% vs 9% in non-irradiated patients; *P* = .0008, Figure 5A). In the absence of additional HR features (metastasis, LCA, STR), *MYC-*MB was compatible with long-term survival (irradiated patients 5-year PFS 63%, Figure 5B); long-term survival was observed in a subset of *MYC*-MB_Grp3_ (Figure 5B). However, no or only marginal improvements in survival were observed following irradiation in patients with ≥1 additional risk factor (Figure 5C; Supplementary Figure 6). Each additional risk factor assessed was individually associated with poorer survival (Supplementary Figure 6); however, these risk factors frequently co-occurred (Figure 5D). Additionally, survival was not significantly different in patients receiving high-dose vs standard-dose chemotherapy (5-year PFS 11% and 24% for high and standard-dose chemotherapy patients, respectively, *P* = .12; Supplementary Figures 5 and 7). In infant patients only, 5-year PFS was 13% and 10% for high and standard-dose chemotherapy patients respectively, *P* = .68).

### Defining Risk in *MYC(N)*-Amplified Patients

**Figure 6.**
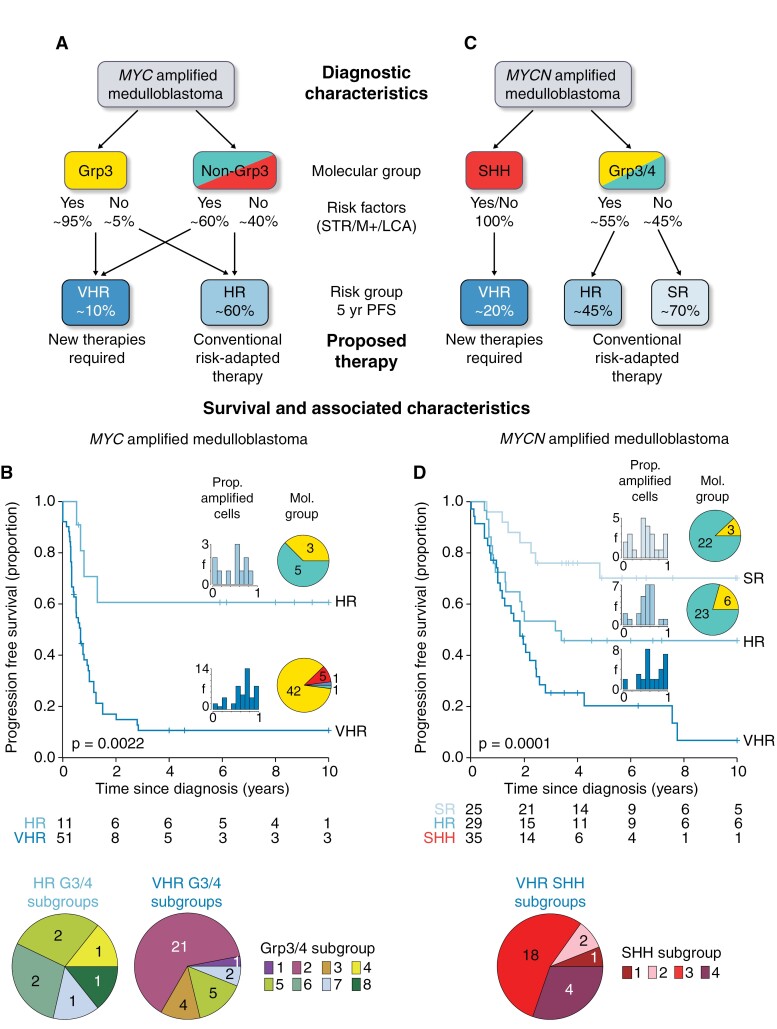
Treatment stratification and survival groups within *MYC*-MB and *MYCN*-MB. (A, C) Suggested stratification for *MYC*-MB and *MYCN*-MB. (B, D) Risk stratification identifies VHR patient groups and groups compatible with longer-term survival. For each treatment group, Kaplan-Meier plots with risk tables are shown, with insets showing additional features of each group—the proportion of amplified cells, molecular group, and subgroup. SR, standard risk; HR, high risk; VHR, very high risk.

Molecular group is critical to assess risk in *MYC*-MB. The presence of additional risk factors (≥1 of STR/M+/LCA) allocates the majority (51/62; 82%) to a VHR group with 11% 5-year PFS (Figure 6A, B). This group is mostly MB_Grp3_ (42/49; 86%), and predominantly MB_Grp3/4_ subgroup 2 (21/34; 62%) and 5 (5/34; 15%). Remaining patients where *MYC* amplification is the sole risk factor are HR (5-year PFS 61%), and mostly MB_Grp4_ (5/8; 63%).

*MYCN*-MB can be assigned into standard risk, HR, and VHR groups (Figure 6C, D). VHR disease was defined by MB_SHH_ (35/89 (39%) patients, 5-year PFS 20%). These were predominantly SHH subgroup 3 (53%; 17/32) and had a higher proportion of amplified cells (*P* = .04; vs standard/HR groups). High-risk disease was defined by MB_Grp3/4_ with positivity for ≥1 of STR/M+/LCA, encompassing 29/89 (33%) patients (5-year PFS 46%). MB_Grp3/4_ patients negative for STR/M+/LCA (25/89 (28%)) were standard risk (5-year PFS, 70%).

## Discussion

*MYC(N)* family amplification is the key molecular biomarker of HR MB in current clinical practice. Our investigation of >150 *MYC(N)*-amplified tumors, drawn from >1600 diagnostic cases, reveals significant clinical and biological heterogeneity within both *MYC* and *MYCN*-amplified disease. Disease molecular group is the primary determinant of their clinical features and interacts with established risk factors and other features to define prognosis (Figure 6). These characteristics must be considered diagnostically and have the potential to immediately impact clinical management. Moreover, to avoid misdiagnosing patients, iFISH for oncogene amplification detection must remain mandatory, since methylation arrays frequently failed to detect amplifications, possibly as a consequence of intra-tumoral heterogeneity.

Our findings define a group of canonical *MYC*-amplified tumors (74%) which are group 3, display other HR features (e.g. LCA, M+, and STR) and have exceptionally poor prognosis (5-year PFS 11%). Noncanonical tumors (non-group 3 or group 3 with *MYC* as the sole HR feature) represent a notable group (26%); experience within our cohort indicates a better prognosis—approximately 60% achieve durable outcomes. Canonical tumors are most commonly subgroup 2 with a high percentage of amplified cells, whereas noncanonical tumors typically comprise other group 3/4 subgroups and have fewer amplified cells. Chromothripsis of chromosome 8 (*MYC*-harboring) and *MYC*-associated fusion genes are common features of all *MYC*-amplified tumors.

*MYCN*-amplified tumors distribute evenly between SHH and group 4; this subdivision is the primary determinant of their clinical behavior. *MYCN*-amplified SHH MB (40%) have dismal outcomes (5-year PFS 20%) and are commonly LCA and/or *TP53*^mut^ (~75% germline); however, these factors do not appear to further influence prognosis. In contrast, *MYCN*-amplified group 4 MB (~50%) more commonly achieve long-term survival, and their prognosis appears equivalent to non-*MYCN*-amplified group 4 MB, with other established factors (e.g. M+) defining their risk. Clinical behavior of rarer *MYCN*-amplified group 3 MB (~10%) appears consistent with group 4. Chromothripsis of chromosome 2 (*MYCN*-harboring) was common, but, in contrast to *MYC*, frequently coinvolved other chromosomes and their transcriptomes contained both intra- and interchromosomal fusion genes.

We proffer a system for risk stratification of *MYC(N)*-amplified tumors (Figure 6), combining molecular groups and other risk factors. Associated markers (subgroup and levels of intra-tumoral amplification) further corroborate and secure these diagnoses. Most importantly, these enable the distinction of VHR tumor groups (canonical *MYC* and *MYCN*-amplified SHH) in which all current therapies (conventional chemotherapy and CSI) are ineffective. Relapse and/or progression are near-universal and novel therapeutic strategies should be urgently considered. Notably, additional driver mutations were rare in both groups (Supplementary Figures 2 and 3) and indirect targeting strategies (e.g. immune and/or cellular therapies, targeting of biological codependencies) will likely be required.^42–44^ In the absence of effective therapies, more palliative strategies could be considered for these VHR groups. We found no evidence to suggest group 4 *MYCN*-amplified and other rarer tumors lying outside these canonical groups share this VHR prognosis, indicating they may be stratified for conventional therapies using established risk markers.^14^

Assembly of this large, retrospective cohort has been essential to understand the clinical behavior of *MYC(N)*-amplified MB. We acknowledge the limitations of its retrospective multicenter nature and the potential to introduce bias in preselected cohorts. Moreover, due to issues of collinearity of HR disease features (Figure 5D) and cohort size, multivariable survival models were not assessable. However, by definition, equivalent investigations will not be achievable in contemporary international clinical trials (typically *n* = 300–400); such cases must therefore be carefully monitored and discussed within a multidisciplinary tumor board setting, based on the available evidence.

In summary, our investigations refine diagnosis and prognostication of *MYC(N)*-amplified MB, allowing the definition of canonical *MYC*-amplified and *MYCN*-amplified SHH patients which are essentially incurable using current therapies and require novel treatment strategies, alongside lower-risk subsets compatible with longer-term survival.

## Supplementary material

Supplementary material is available online at *Neuro-Oncology* (https://academic.oup.com/neuro-oncology).

noae178_suppl_Supplementary_Figures

## Data Availability

Data is available from the authors on reasonable request.
