# Supplementary material for: Molecular and clinical heterogeneity within MYC-family amplified medulloblastoma is associated with survival outcomes: A multicenter cohort study
Source: Neuro Oncol. 2024 Oct 8;27(1):222–36. doi: 10.1093/neuonc/noae178 (PMC11726341; doi:10.1093/neuonc/noae178)
Supplement: noae178_suppl_Supplementary_Figures [file noae178_suppl_supplementary_figures.zip › Supplementary Figure Legends_final.docx]

**Supplementary Figure 1. OS and PFS for *MYC*-amplified and *MYCN*-amplified patients are not significantly different. A.** OS/PFS for all *MYC*-amplified patients. **B.** OS/PFS for *MYC*-amplified patients aged under 3 at diagnosis. **C.** OS/PFS for *MYCN*-amplified patients receiving cranio-spinal irradiation. At risk tables and p values from logrank tests are shown.

**Supplementary Figure 2.** **The mutational landscape of *MYC*-amplified medulloblastoma.** Oncoplot shows distributions of commonly mutated genes in *MYC* (n=22) amplified medulloblastomas with available panel sequencing data. Missing data are shown gray.

**Supplementary Figure 3. The mutational landscape of *MYCN*-amplified medulloblastoma.** **A.** Oncoplot shows distributions of commonly mutated genes in *MYCN* (n=31) amplified medulloblastomas with available panel sequencing data. **B.** The clinico-molecular features of an extended cohort of SHH medulloblastomas with *MYCN* amplification and *TP53* mutation status (n=35). Missing data are shown gray.

**Supplementary Figure 4. Characterization of chromothripsis and validation of fusion transcripts in *MYC* and *MYCN*-amplified medulloblastoma. A.** Circos plots show RNA fusion landscape of a *MYCN*-amplified tumor’s four chromothriptic chromosomes (1,2,7 and 8) and two *MYC*-amplified tumors with chromothripsis of chromosome 8. SNP6-derived copy number profiles of four chromothriptic chromosomes from the *MYCN*-amplified tumor and chromothripsis in chromosome 8 for two *MYC*-amplified tumors are also shown*.* **B. (i)** Region of chromosome 2 surrounding the *MYCN* locus in a *MYCN*_GRP3_, tumor showing amplification of multiple regions and fusion transcripts detected by RNA-seq between genes on these amplified regions **(ii)** confirmation of two of these transcripts by PCR (primers and fusion junction (vertical line) indicated in red) and Sanger sequencing. **C. (i)** Frequency and distribution of *PVT* fusion products in 7 *MYC*-amplified tumors **(ii)** Confirmation of selected fusion transcripts by PCR and Sanger sequencing.

**Supplementary Figure 5.** **Survival of patients with *MYC* and *MYCN*-amplified tumors.** **A, B**. Summary tables of univariable Cox proportional hazard models shows hazard ratio (HR), 95% confidence intervals (95% CI), p value and the number of assessable tumors. **C, D**. Survival of MB_SHH_-*MYCN*-amplified medulloblastoma by *TP53* and *GLI1/2* amplification status. **E.** Survival of MB_Grp3_-*MYC* amplified medulloblastoma stratified by infant status. Kaplan-Meier plots and at-risk tables are shown.

**Supplementary Figure 6. Cranio-spinal irradiation is ineffective in *MYC*-amplified tumors with other high-risk disease features. A-C.** Kaplan-Meier plots and at-risk tables stratified by receipt of cranio-spinal irradiation are shown for patients positive for LCA disease, metastatic disease and sub-total resection respectively. Where appropriate, molecular group is indicated by filled circles adjacent to censor points for survivors with PFS ≥ 4 years; molecular group is shown as inset pie charts; certain MYC-amplified tumors lacked molecular group information and were omitted from pie charts.

**Supplementary Figure 7. High-dose chemotherapy does not improve outcomes in *MYC* and *MYCN*-amplified tumors. A-C.** Kaplan-Meier plots and at-risk tables stratified by receipt of high and standard dose chemotherapy are shown for all *MYC*-amplified patients, all *MYCN-*amplified patients and *MYC*-amplified infant patients.
